# Extracellular Lactic Acidosis of the Tumor Microenvironment Drives Adipocyte-to-Myofibroblast Transition Fueling the Generation of Cancer-Associated Fibroblasts

**DOI:** 10.3390/cells12060939

**Published:** 2023-03-20

**Authors:** Elena Andreucci, Bianca Saveria Fioretto, Irene Rosa, Marco Matucci-Cerinic, Alessio Biagioni, Eloisa Romano, Lido Calorini, Mirko Manetti

**Affiliations:** 1 Department of Experimental and Clinical Biomedical Sciences “Mario Serio”, Section of Experimental Pathology and Oncology, University of Florence, 50134 Florence, Italy; 2 Department of Experimental and Clinical Medicine, Section of Anatomy and Histology, University of Florence, 50134 Florence, Italy; 3 Department of Experimental and Clinical Medicine, Section of Internal Medicine, University of Florence, 50134 Florence, Italy; 4 Department of Experimental and Clinical Medicine, Imaging Platform, University of Florence, 50134 Florence, Italy

**Keywords:** adipocytes, adipose-derived stem cells, cell differentiation, extracellular acidosis, lactate, myofibroblasts, cancer-associated fibroblasts, breast cancer cells

## Abstract

Lactic acidosis characterizes the tumor microenvironment (TME) and is involved in the mechanisms leading to cancer progression and dissemination through the reprogramming of tumor and local host cells (e.g., endothelial cells, fibroblasts, and immune cells). Adipose tissue also represents a crucial component of the TME which is receiving increasing attention due to its pro-tumoral activity, however, to date, it is not known whether it could be affected by the acidic TME. Now, emerging evidence from chronic inflammatory and fibrotic diseases underlines that adipocytes may give rise to pathogenic myofibroblast-like cells through the adipocyte-to-myofibroblast transition (AMT). Thus, our study aimed to investigate whether extracellular acidosis could affect the AMT process, sustaining the acquisition by adipocytes of a cancer-associated fibroblast (CAF)-like phenotype with a pro-tumoral activity. To this purpose, human subcutaneous adipose-derived stem cells committed to adipocytes (acADSCs) were cultured under basal (pH 7.4) or lactic acidic (pH 6.7, 10 mM lactate) conditions, and AMT was evaluated with quantitative PCR, immunoblotting, and immunofluorescence analyses. We observed that lactic acidosis significantly impaired the expression of adipocytic markers while inducing myofibroblastic, pro-fibrotic, and pro-inflammatory phenotypes in acADSCs, which are characteristic of AMT reprogramming. Interestingly, the conditioned medium of lactic acidosis-exposed acADSC cultures was able to induce myofibroblastic activation in normal fibroblasts and sustain the proliferation, migration, invasion, and therapy resistance of breast cancer cells in vitro. This study reveals a previously unrecognized relationship between lactic acidosis and the generation of a new CAF-like cell subpopulation from adipocytic precursor cells sustaining tumor malignancy.

## 1. Introduction

Extracellular acidosis characterizes the microenvironment of most solid tumors which generally show a pH ranging from 6.4 to 7.0 [1]. The acidification of the tumor microenvironment (TME) is indeed the direct consequence of the high glycolytic metabolism of cancer cells, which prefer glycolysis instead of phosphorylative oxidation even in presence of oxygen (Warburg effect), with a final advantage in cell proliferation but accompanied by an overproduction and subsequent release of lactic acid in the extracellular milieu [2,3]. Besides that, the impaired lymphatic circulation and the high interstitial pressure typical of solid cancer tissues further exacerbate the phenomenon [4].

The acidic TME has been recognized as a hallmark of cancer and a crucial contributor to tumor progression. Notably, extracellular acidosis has been demonstrated to reprogram both tumor and host stromal cellular components toward disease advancement, actively participating in every step of cancer dissemination [5,6,7]. Much evidence to date has revealed the effects of the acidic TME on cancer cells, which, once adapted to such environmental conditions, gain an extremely plastic phenotype endowed with an increased ability to invade the surrounding tissues, enter into and survive in the blood/lymphatic circulation (anoikis resistance), and evade immune surveillance systems, as well as resist chemotherapy, radiation therapy, immunotherapy, and molecularly targeted therapies, overall accounting for an increased metastatic potential [5,8,9,10].

The acidic TME exerts pro-tumoral effects by also acting on non-tumor cellular components, either within or surrounding the tumor mass, or even at distant sites—in a sort of paracrine/endocrine way, involving, for instance, extracellular vesicles that can travel through all body fluids, conveying oncogenic bioactive molecules and potentially preparing every tissue for metastatic colonization [11]. Indeed, the ability of extracellular acidosis to drive the pro-tumoral reprogramming of fibroblasts [12,13,14], endothelial cells [15,16], and immune cells [17,18,19,20] has been extensively demonstrated. To date, no evidence has been collected instead on the possible effects of extracellular acidosis on the adipocytic cell compartment and their possible switching through the adipocyte-to-myofibroblast transition (AMT). Notably, very recent findings underline the importance of AMT, a process by which pre-adipocytes/adipocytes transdifferentiate into pathogenic myofibroblast-like cells, observed in systemic sclerosis and other fibrotic diseases, where it was deemed as relevant in the formation of the pro-fibrotic myofibroblasts responsible for excessive extracellular matrix synthesis/deposition and remodeling [21,22,23,24]. Adipocytes and, more precisely, cancer-associated adipocytes (CAAs) are then being extensively investigated, and evidence supports their active contribution to cancer progression, especially in mammary tumors [20,25,26,27,28,29], where they represent 90% of the breast tissue [30] and were found to give rise to the generation of cancer-associated fibroblast (CAF)-like cells with pro-tumoral activity [31]. To date, it has never been explored whether extracellular lactic acidosis might favor AMT.

Here, indeed, we demonstrated for the first time that lactic acidosis promotes the AMT process, resulting in the generation of myofibroblast/CAF-like cells that in turn sustain proliferation, migration, and invasion, as well as the therapy resistance of breast cancer cells.

## 2. Materials and Methods

### 2.1. Cell Culture and Treatment

Three lines of normal human subcutaneous adipose-derived stem cells (ADSCs) purchased from Lonza (catalog no. PT-5006; Lonza, Basel, Switzerland) were maintained in ADSC complete medium (ADSC Growth Medium BulletKit; catalog no. PT-4505; Lonza) at 37 °C in a 5% CO_2_ incubator. To commit ADSCs to adipocytes, cells at low passage (passages 2–4) and at 90% confluence were seeded in Petri dishes and incubated for 10 days in a preadipocyte differentiation medium (PGM-2 Preadipocyte Growth Medium-2 BulletKit; catalog no. PT-8002; Lonza) containing PBM-2 basal medium (catalog no. PT-8202; Lonza) and PGM-2 SingleQuots supplements (catalog no. PT-9502; Lonza). The obtained adipocyte-committed ADSCs (acADSCs) were then cultured for another 3 days under different conditions to obtain three different experimental points, namely basal acADSCs, acidic acADSCs, and transforming growth factor β1 (TGFβ1)-treated acADSCs. Basal acADSCs were cultured in PBM-2 basal medium with 2% fetal bovine serum (FBS) at pH 7.4, acidic acADSCs were subjected to chemically induced acidosis by the direct administration of 1 N hydrogen chloride (HCl) in PBM-2 medium with 2% FBS to reach pH 6.7 in the presence of 10 mM lactate (Sigma-Aldrich, Milan, Italy), while TGFβ1-treated acADSCs were incubated in PBM-2 medium with 2% FBS (pH 7.4) and 10 ng/mL recombinant human TGFβ1 (PeproTech, Rocky Hill, NJ, USA). pH 6.7 was chosen as the representative acidic condition following preliminary experiments where a pH range from 7.4 to 6.4 was assessed in our cell culture model. pH was monitored using an Orion 520A1 pH meter (Thermo Fisher Scientific, Waltham, MA, USA). An additional experimental condition of acADSCs grown for 3 days in the basal medium at pH 7.4 in the presence of 10 mM lactate was used as an internal control in the initial experiments on AMT. Phase-contrast photomicrographs of acADSCs were captured under a Leica inverted microscope (Leica Microsystems, Mannheim, Germany). Cells with intracytoplasmic lipid droplets were counted in 10 randomly chosen microscopic high-power fields (hpf; 40× original magnification) per sample by two independent observers (B.S.F. and M.M.) who were blinded with regard to the sample classification. The final result was the mean of the two different observations for each sample. For selected experiments, conditioned media from basal and acidic acADSCs (basal acADSC-cm and acidic acADSC-cm, respectively) were collected after maintaining cells in fresh PBM-2 basal medium supplemented with 2% FBS for an additional 3 days.

Three lines of normal human dermal fibroblasts (NHDFs) purchased from Sigma-Aldrich (catalog no. C-12302) and cultured in Dulbecco’s modified Eagle medium (DMEM), 4.5 g/L glucose (Euroclone, Milan, Italy) supplemented with 2 mM L-glutamine and 10% FBS were used for the experimental procedures between the third and seventh passages.

MCF7 and MDA-MB-231 breast cancer cells were obtained from American Type Culture Collection (ATCC) and cultured in DMEM 4.5 g/L glucose supplemented with 10% FBS. In selected experiments, MCF7 and MDA-MB-231 were treated for 72 h with 0.1 µM and 1 µM doxorubicin (MedChemExpress, Sollentuna, Sweden), respectively.

### 2.2. Annexin V/Propidium Iodide Flow Cytometer Assay

ADSCs were seeded into 6-well plates until 90% confluence, committed to adipocyte differentiation for 10 days, and subsequently grown for 72 h under basal (pH 7.4) or lactic acidic (pH 6.7, 10 mM lactate) conditions. Culture media were collected and acADSCs were harvested with Accutase (Euroclone), and subsequently collected in flow cytometer tubes. After 5 min centrifugation at 300× *g*, cell pellets were incubated for 15 min at 4 °C in the dark with 100 μL of annexin binding buffer (100 mM HEPES, 140 mM NaCl, 25 mM CaCl_2_, pH 7.4), 1 μL of 100 μg/mL propidium iodide (PI; Sigma-Aldrich) working solution, and 3 μL annexin V APC-conjugated (Immunotools, Friesoythe, Germany). Each sample was added with annexin binding buffer to reach 500 μL volume/tube. Samples were then analyzed at BD FACSCanto II (BD Biosciences, Milan, Italy). Cellular distribution depending on annexin V and/or PI positivity allowed the measurement of the percentage of viable cells (annexin V^−^ and PI^−^), early apoptosis (annexin V^+^ and PI^−^), late apoptosis (annexin V^+^ and PI^+^), and necrosis (annexin V^−^ and PI^+^). A minimum of 10,000 events were collected.

### 2.3. MTT Assay

After 1 × 10^4^ ADSCs were seeded into a 96-well plate and committed to adipocyte differentiation for 10 days, the obtained acADSCs were grown for 72 h under basal (pH 7.4) or lactic acidic (pH 6.7, 10 mM lactate) conditions. Cells were subsequently incubated for 2 h at 37 °C in the dark with 0.5 mg/mL MTT-containing medium without phenol red. MTT was removed and cells were lysed in 100 µL dimethyl sulfoxide (DMSO). Absorbance values were recorded at 595 nm with an automatic plate reader (Bio-Rad, Hercules, CA, USA). Similarly, 0.5 × 10^4^ MCF7 or MDA-MB-231 cells were seeded into a 96-well plate and grown in basal acADSC-cm or acidic acADSC-cm for 72 h or 96 h as specified in the results section and figure legends. Following the 2 h incubation at 37 °C in 0.5 mg/mL MTT-containing medium in the absence of phenol red, cells were lysed in 100 µL DMSO, and 595 nm absorbance values were measured with the automatic plate reader.

### 2.4. RNA Isolation and Quantitative Real-Time Polymerase Chain Reaction (qPCR)

Total RNA was prepared from acADSCs using Tri Reagent (Merck Life Science, Milan, Italy), agarose gel checked for integrity, and quantified with the NanoDrop 8000 Spectrophotometer (Thermo Fisher Scientific). In selected experiments, RNA was also purified from NHDFs cultured in basal acADSC-cm or acidic acADSC-cm for 24 h. Reverse transcription was performed with the iScript cDNA Synthesis Kit (Bio-Rad, Milan, Italy) according to the manufacturer’s instructions. For gene expression quantification, SYBR Green Real-Time PCR was performed using the StepOnePlus Real-Time PCR System (Applied Biosystems, Milan, Italy) with melting curve analysis. Predesigned oligonucleotide primer pairs were employed (QuantiTect Primer Assay; Qiagen, Hilden, Germany). The assay IDs are shown in Table 1. The PCR mixture was composed of 1 μL cDNA, 0.5 μM sense, and antisense primers, 10 μL 2× QuantiTect SYBR Green PCR Master Mix containing SYBR Green I dye, ROX passive reference dye, HotStarTaq DNA Polymerase, dNTP mix, and MgCl_2_ (Qiagen). Amplification was performed according to a standard protocol recommended by the manufacturer. Non-specific signals produced by primer dimers or genomic DNA were excluded by dissociation curve analysis, non-template controls, and samples without enzyme in the reverse transcription step. In all samples, 18S ribosomal RNA (Hs_RRN18S_1_SG; catalog no. QT00199367; Qiagen) was measured as an endogenous control to normalize the amounts of loaded cDNA. Differences were calculated with the threshold cycle (Ct) and comparative Ct method for relative quantification.

### 2.5. Western Blotting

acADSCs, grown for 3 days under basal (pH 7.4) or lactic acidic (pH 6.7, 10 mM lactate) conditions were lysed in radioimmunoprecipitation assay (RIPA) lysis buffer (Millipore by Sigma-Aldrich) added with Pierce Protease Inhibitor Tablets (Thermo Fisher Scientific) for protein isolation. The protein concentration was measured with Bradford reagent (Millipore by Sigma-Aldrich). After the addition of the Laemmli sample buffer (Bio-Rad) and β-mercaptoethanol, equal amounts of proteins were boiled at 100 °C for 5 min, electrophoresed on precast polyacrylamide gels (4-15% Mini-Protean TGX Gels; Bio-Rad), and blotted onto nitrocellulose membranes (Bio-Rad). The membranes were blocked for 30 min at room temperature in 5% *w*/*v* milk buffer (5% *w*/*v* non-fat dried milk, 50 mM Tris, 200 mM NaCl, 0.2% Tween-20) and subsequently incubated overnight at 4 °C with the following primary antibodies diluted in 5% *w*/*v* milk buffer: rabbit monoclonal anti-fatty acid-binding protein 4 (FABP4) (1:1000 dilution; catalog no. ab92501; Abcam, Cambridge, UK), mouse monoclonal anti-adiponectin (1:1000 dilution; catalog no. ab22554; Abcam), rabbit polyclonal anti-perilipin-1 (1:500 dilution; catalog no. ab3526; Abcam), mouse monoclonal anti-α-smooth muscle actin (α-SMA) (1:300 dilution; catalog no. ab7817; Abcam), rabbit monoclonal anti-COL1A1 (1:1000 dilution; catalog no. #39952; Cell Signaling Technology, Danvers, MA, USA), and goat polyclonal anti-GPR81 (1:1000 dilution; catalog no. ab106942; Abcam). Mouse monoclonal anti-glyceraldehyde 3-phosphate dehydrogenase (GAPDH) (1:5000 dilution; catalog no. ab8245; Abcam) and rabbit polyclonal anti-α-actinin (1:1000 dilution; catalog no. #3134; Cell Signaling Technology) antibodies were used for normalization. The immunoblots were washed three times in Tris-buffered saline (Bio-Rad) with 0.1% Tween-20, and then incubated for 1 h at room temperature with HRP-conjugated secondary antibodies (Cell Signaling Technology). The proteins were visualized by an enhanced chemiluminescence method (Clarity Western ECL Substrate; Bio-Rad) and analyzed by ChemiDoc Touch Imaging System (Bio-Rad). Band intensities were quantified using the free-share ImageJ software (NIH, Bethesda, MD, USA; online at http://rsbweb.nih.gov/ij, accessed on 23 May 2022) and values were normalized to GAPDH or α-actinin, as needed.

### 2.6. Immunofluorescence

For immunofluorescence microscopy, 2.5 × 10^5^ ADSCs were seeded onto glass coverslips in 6-well plates and committed to adipocyte differentiation for 10 days. The resulting acADSCs were grown for 72 h under basal (pH 7.4) or lactic acidosis (pH 6.7, 10 mM lactate) conditions. Cells were then fixed for 30 min at 4 °C with 3.7% buffered paraformaldehyde and permeabilized for 15 min with PBS 0.1% Triton X-100 at room temperature. Slides were then blocked with 1% bovine serum albumin in PBS for 1 h at room temperature, and finally incubated overnight at 4 °C with the following primary antibodies: rabbit polyclonal anti-perilipin-1 (1:80 dilution; catalog no. ab3526; Abcam), mouse monoclonal anti-α-SMA (1:100 dilution; catalog no. ab7817; Abcam), mouse monoclonal anti-adiponectin (1:100 dilution; catalog no. ab22554; Abcam), and rabbit monoclonal anti-COL1A1 (1:300 dilution; catalog no. #39952; Cell Signaling Technology). Irrelevant isotype-matched and concentration-matched mouse and rabbit IgG (Sigma-Aldrich) were used as negative controls. The day after, cells were incubated for 45 min at room temperature in the dark with Alexa Fluor-488-conjugated and Rhodamine Red-X-conjugated secondary antibodies at 1:200 dilution (Invitrogen, Carlsbad, CA, USA). Following nuclei staining with 4′,6-diamidino-2-phenylindole (DAPI; Thermo Fisher Scientific) for 10 min at room temperature in the dark, the immunostained cells were mounted onto glass slides and visualized at the Leica DM4000 B microscope (Leica Microsystems). Fluorescence images were captured with a Leica DFC310 FX 1.4-megapixel digital color camera equipped with the Leica software application suite LAS V3.8 (Leica Microsystems). Immunostained cells were counted in 10 randomly chosen microscopic hpf (40× original magnification) per sample. Only the cells with well-defined DAPI-positive nuclei were counted. Counting was performed by two independent observers (I.R. and M.M.) who were blinded with regard to the sample classification. The final result was the mean of the two different observations for each sample.

### 2.7. Colony Assay

The 0.2 × 10^3^ MCF7 or MDA-MB-231 cells were seeded into 6-well plates and allowed to grow in the presence of the basal acADSC-cm or acidic acADSC-cm for 10 days. Formed colonies were then fixed in 3.7% paraformaldehyde and stained with a crystal violet solution.

### 2.8. Migration and Invasion Assays

Migration and invasion assays on MCF7 and MDA-MB-231 cells were performed following a 24 h treatment with basal acADSC-cm or acidic acADSC-cm. For the migration assay, 12 mm diameter Millicell cell culture inserts with 8 µm diameter-pore polycarbonate filters (Sigma-Aldrich) were placed into 24-well plates, and 5 × 10^4^ cancer cells were seeded in the upper compartment and allowed to migrate for 6 h without any FBS gradient toward the lower compartment. Following a 1 h fixation in methanol at 4 °C, non-invading cells on the upper side of the filters were mechanically wiped off with a cotton swab, while invasive cells on the lower side of the filters were stained with Diff-Quik dye (BD Biosciences). Cells were then visualized and counted using an optical microscope. For the invasion assay, an analog experimental procedure was performed with the difference that the polycarbonate filters of the inserts were pre-coated overnight with 0.25 µg/µL Matrigel (Corning by Sigma-Aldrich) before cell seeding.

### 2.9. Statistical Analysis

All data were obtained based on at least three independent experiments and analyzed with GraphPad Prism 8 software. After assessing the normality of data by the Kolmogorov–Smirnov test, statistical analysis between the two groups was performed using an unpaired Student’s *t*-test. In the case of the comparative analysis of three groups, one-way analysis of variance (ANOVA) was performed followed by Tukey’s post hoc test. Values are presented as the mean of independent experiments ± standard error of the mean (SEM). Values of *p* < 0.05 were considered statistically significant.

## 3. Results

### 3.1. Lactic Acidosis Induces an Adipocyte-to-Myofibroblast Transition in Adipocyte-Committed Subcutaneous Adipose-Derived Stem Cells

**Figure 1:**
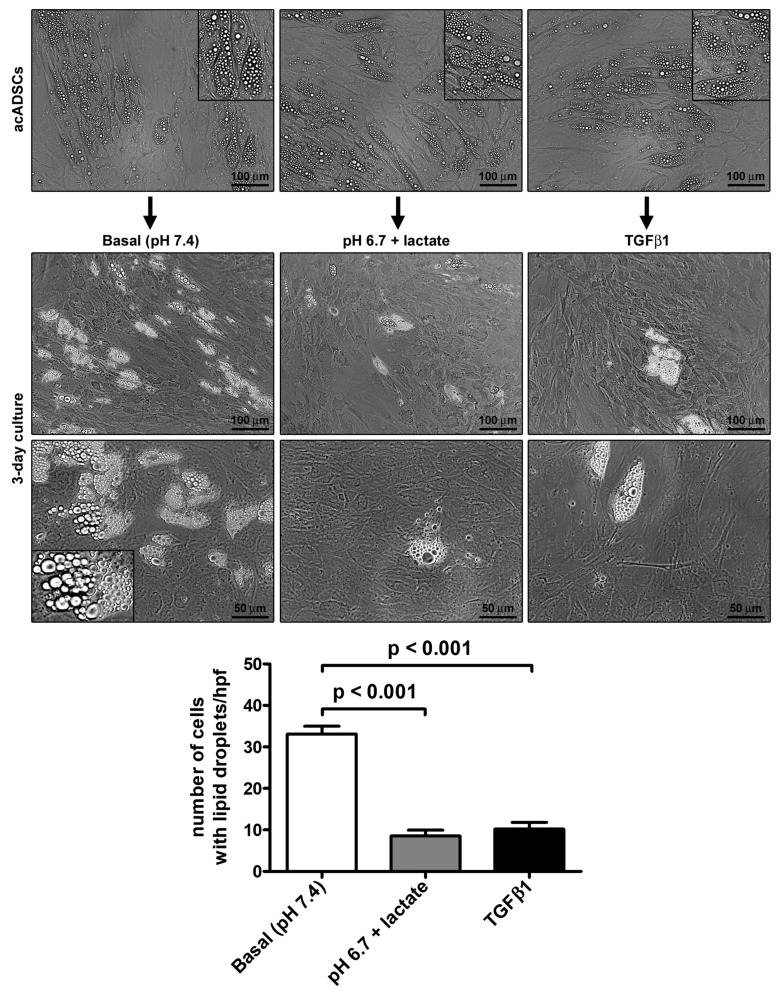
Representative phase-contrast photomicrographs of adipocyte-committed adipose-derived stem cells (acADSCs) cultured under basal conditions (pH 7.4), lactic acidosis (pH 6.7 + lactate), or in the presence of 10 ng/mL TGFβ1 for 3 days. Insets are higher magnification views of the respective panels, displaying prominent intracytoplasmic lipid droplets. Scale bar: 100 µm (upper and middle panels), 50 µm (lower panels). Bars represent the mean ± SEM of the number of cells with intracytoplasmic lipid droplets/high-power field (hpf). Values of *p* were determined by Tukey’s test.

Preliminary experiments performed on ADSCs allowed us to choose pH 6.7, out of a pH range from 7.4 to 6.4, as the representative acidic condition for our experimental model, ensuring that cell viability under the conditions of pH 6.7 was not impaired (Supplementary Figure S1). By cultivating acADSCs under basal conditions (pH 7.4) or lactic acidosis (pH 6.7 + lactate), we observed that the acidic microenvironment induced deep morphologic changes in these cells. Indeed, compared to basal conditions, acADSCs grown in a lactic acidic medium showed a substantial loss of intracytoplasmic lipid droplets while displaying a mesenchymal-like elongated shape (Figure 1). This observation suggests that, under such acidic conditions, acADSCs undergo phenotypic remodeling toward a mesenchymal phenotype. The AMT process has already been identified in several fibrotic diseases as the mechanism by which pre-adipocytes/adipocytes transdifferentiate into myofibroblasts, and TGFβ1 was found to induce such a phenotypic switch [22,23,24]. Thereby, acADSCs cultured in standard pH in the presence of 10 ng/mL TGFβ1 represented a positive control of AMT. In fact, acADSCs treated with TGFβ1, compared to those grown under basal conditions, displayed a mesenchymal-like, elongated shape—even further marked compared to lactic acidosis—accompanied by a considerable decrease in intracytoplasmic lipid droplets. As shown in Figure 1, the number of cells containing cytoplasmic lipid droplets was significantly decreased in both acADSCs cultured under lactic acidosis and those stimulated with TGFβ1 compared to cultures maintained in basal (pH 7.4) medium.

**Figure 2:**
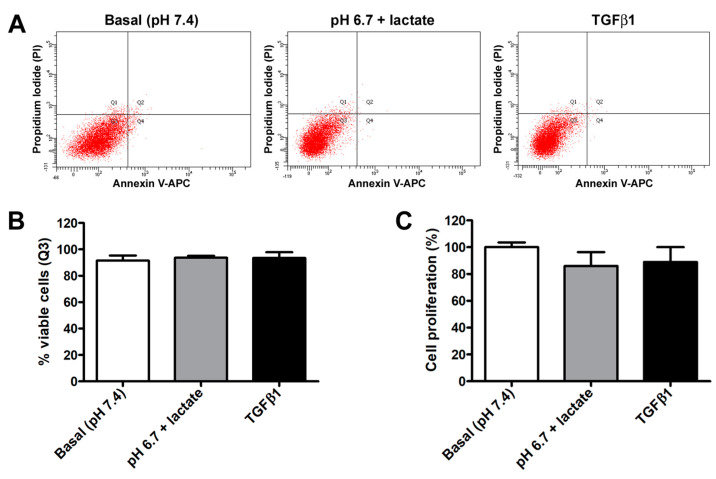
Representative flow cytometer plots of annexin V/propidium iodide (PI) staining (**A**) with cell viability quantification (**B**), and proliferation chart (**C**) of adipocyte-committed adipose-derived stem cells (acADSCs) cultured for 3 days under basal conditions (pH 7.4), lactic acidosis (pH 6.7 + lactate), or in the presence of 10 ng/mL TGFβ1. Proliferation of acADSCs at basal conditions was set to 100%, and the other results are normalized to this value. Bars represent the mean ± SEM of three independent experiments (*n* = 3 replicates each for Annexin V/PI assay, *n* = 10 replicates each for proliferation assay) from three cell lines. No statistical difference among the three experimental groups was detected for both percentages of viable cells (**B**) and cell proliferation (**C**) by one-way ANOVA.

In contrast, we did not observe any significant variation in acADSC viability or proliferation when grown in basal conditions, lactic acidosis, or in the presence of TGFβ1 (Figure 2A–C). Indeed, annexin V/PI assay revealed that the percentage of viable cells at 72 h was approximately 90% in all experimental conditions (Figure 2A,B). Besides viability, cell proliferation evaluated by MTT assay was also not altered, despite a slight but non-significant decreasing tendency observed in acADSCs subjected to lactic acidosis or TGFβ1 treatment (Figure 2C).

**Figure 3:**
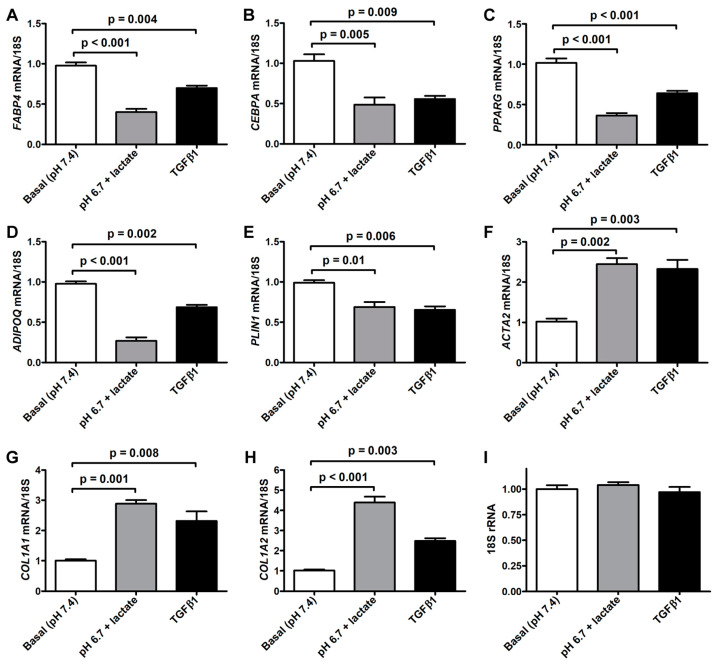
Quantitative real-time PCR analysis for the expression of the adipocytic genes *FABP4* (**A**), *CEBPA* (**B**), *PPARG* (**C**), *ADIPOQ* (**D**), and *PLIN1* (**E**) and the myofibroblastic genes *ACTA2* (**F**), *COL1A1* (**G**), and *COL1A2* (**H**) in adipocyte-committed adipose-derived stem cells grown for 3 days under basal conditions (pH 7.4), lactic acidosis (pH 6.7 + lactate), or in the presence of 10 ng/mL TGFβ1. The basal level of each gene expression was set to 1, and the other results are normalized to this value. 18S ribosomal RNA (rRNA) was used as the reference gene. The expression levels of 18S rRNA were stable under the three different experimental conditions (**I**). Bars represent the mean ± SEM of three independent experiments (*n* = 3 replicates each) from three cell lines. Values of *p* were determined by Tukey’s test.

The morphologic changes displayed by acADSCs subjected to the lactic acidic microenvironment were accompanied by the loss of the expression of typical adipogenic/adipocytic markers and a concomitant increase in the expression of myofibroblast markers (Figure 3A–H). Quantitative real-time PCR analysis revealed that the mRNA expression of the adipocyte-related genes *FABP4*, *CEBPA* (i.e., gene encoding C/EBPα), *PPARG* (i.e., gene encoding PPARγ), and *PLIN1* (i.e., gene-encoding perilipin-1) was almost halved in acADSCs subjected to the lactic acidic microenvironment, while a further decrease was observed for *ADIPOQ* (i.e., gene encoding adiponectin) mRNA expression levels (Figure 3A–E). On the contrary, lactic acidosis increased the expression levels of typical myofibroblast-related genes, such as *ACTA2* (i.e., gene encoding α-SMA), *COL1A1*, and *COL1A2*, which were 2.5-fold, 3-fold, and 4-fold increased, respectively, compared to the basal conditions (Figure 3F–H). As expected, the treatment with TGFβ1, compared to the basal condition, significantly reduced the expression levels of all the adipocyte-related genes tested and concomitantly boosted the expression of myofibroblastic genes (Figure 3A–H). The expression levels of the 18S rRNA reference gene were stable under the three different experimental conditions (Figure 3I).

**Figure 4:**
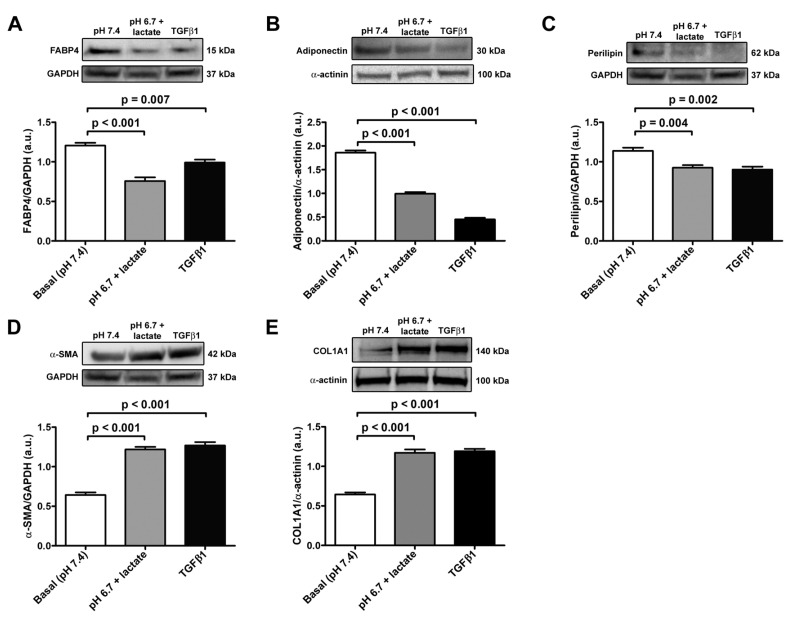
Representative bands of Western blot analysis and the relative quantification charts of the expression of the adipocytic markers FABP4 (**A**), adiponectin (**B**), and perilipin-1 (**C**) and the myofibroblastic markers α-SMA (**D**) and COL1A1 (**E**) in adipocyte-committed adipose-derived stem cells grown for 3 days under basal conditions (pH 7.4), lactic acidosis (pH 6.7 + lactate), or in the presence of 10 ng/mL TGFβ1. GAPDH and α-actinin were measured as loading controls for normalization. Molecular weight values (kDa) are indicated. Bars represent the mean ± SEM of optical density in arbitrary units (a.u.). Values of *p* were determined by Tukey’s test.

The data obtained in real-time PCR were validated with Western blot analysis, which confirmed the trend of the expression of adipocyte/myofibroblast markers (Figure 4A–E). Briefly, we observed a significant decrease in FABP4, adiponectin, and perilipin-1 protein expression both in acADSCs grown under lactic acidosis and in those treated with TGFβ1 compared to basal conditions (Figure 4A–C). On the contrary, the protein expression levels of the myofibroblastic markers α-SMA and COL1A1 were significantly increased by approximately 2-fold in acADSCs cultured in a lactic acidic medium, as well as in the presence of TGFβ1, compared to those grown under basal conditions (Figure 4D,E).

**Figure 5:**
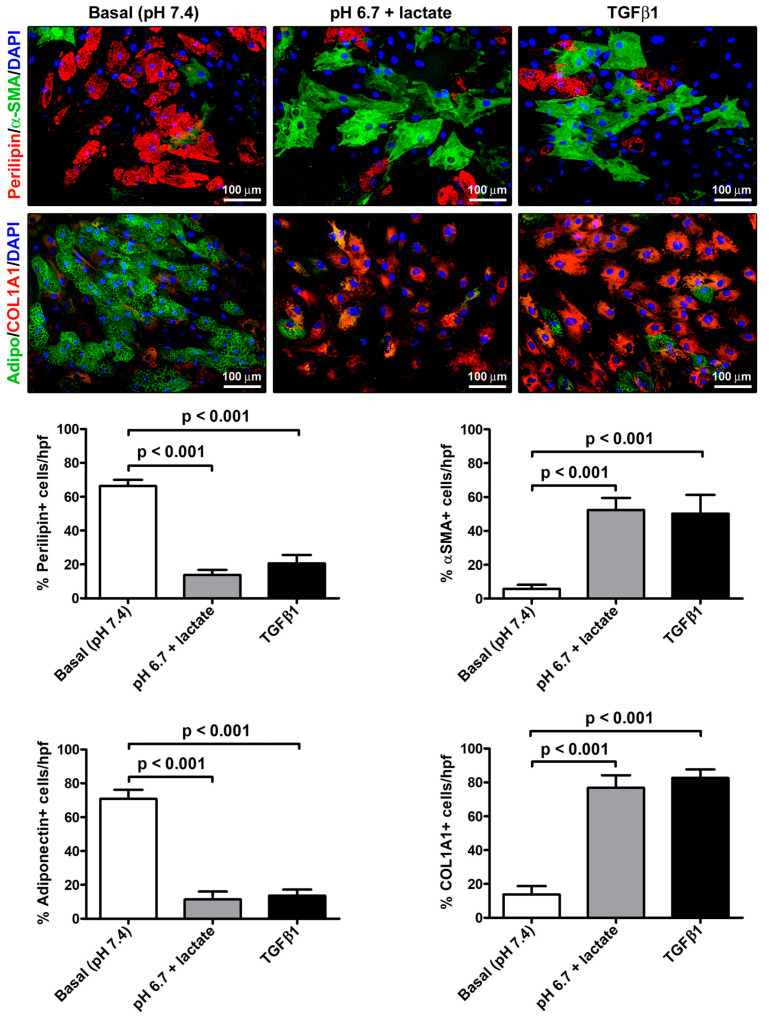
Immunofluorescence analysis for the expression of the adipocytic markers perilipin-1 (red, upper panels) and adiponectin (green, lower panels), and the myofibroblastic markers α-SMA (green, upper panels) and COL1A1 (red, lower panels) in adipocyte-committed adipose-derived stem cells grown for 3 days under basal (pH 7.4) conditions, lactic acidosis (pH 6.7 + lactate), or in the presence of 10 ng/mL TGFβ1. Nuclei are stained blue with DAPI. Scale bar: 100 µm. Bars represent the mean ± SEM of the percentage of immunopositive cells/high-power field (hpf). Values of *p* were determined by Tukey’s test.

The immunofluorescence analysis further strengthened the evidence of the AMT process that the acADSCs underwent upon exposure to an acidic microenvironment (Figure 5). Indeed, we observed a prominent loss of perilipin-1-coated cytoplasmic lipid droplets and the accumulation of intracellular COL1A1 and expression of α-SMA-positive stress fibers characteristic of myofibroblasts in acidic cell cultures, which was similar to that found in cultures stimulated with TGFβ1 (Figure 5). In particular, the percentages of perilipin-1-positive and adiponectin-positive cells/hpf were approximately three times decreased in acADSCs grown under lactic acidosis, as well as following TGFβ1 treatment, compared to those maintained in basal (pH 7.4) medium (Figure 5). Under the same experimental conditions (i.e., lactic acidosis or TGFβ1), the percentages of α-SMA-positive and COL1A1-positive cells/hpf were significantly increased compared to those under basal conditions (Figure 5).

Of note, acADSCs grown for 3 days in a pH 7.4 medium in the presence of 10 mM lactate did not show any relevant change in cell morphology, as well as in the expression of perilipin-1, adiponectin, α-SMA, and COL1A1 compared to the cells grown under basal conditions (Supplementary Figure S2A,B). Therefore, the exposure of acADSCs to lactate alone, without the acidification of the culture medium, was not able to induce the AMT process. As far as the expression of the lactate receptor GPR81 is concerned, no difference was detected between basal acADSCs and those grown under lactic acidosis or at pH 7.4 in the presence of 10 mM lactate (Supplementary Figure S3).

### 3.2. Lactic Acidosis Induces an Adipocyte-to-Myofibroblast Transition in Adipocyte-Committed Subcutaneous Adipose-Derived Stem Cells Characterized by a Pro-inflammatory Phenotype

**Figure 6:**
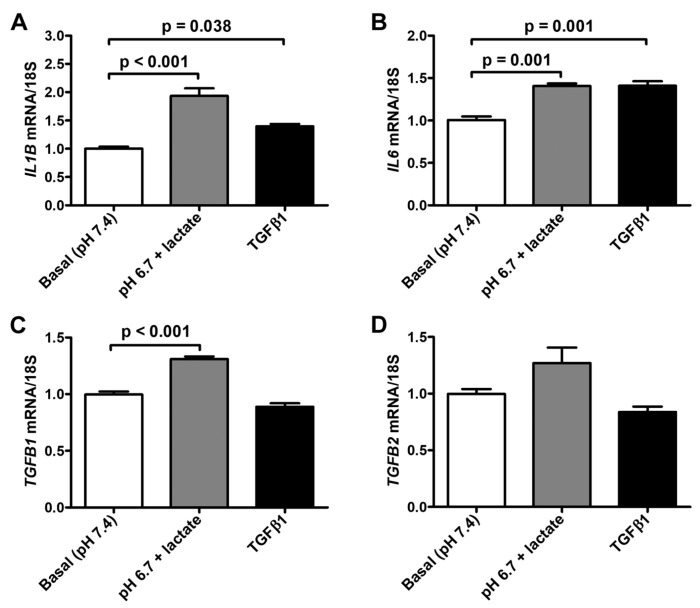
Quantitative real-time PCR analysis for the expression of the pro-inflammatory *IL1B* (**A**) and *IL6* (**B**) genes and the pro-fibrotic *TGFB1* (**C**) and *TGFB2* (**D**) genes in adipocyte-committed adipose-derived stem cells grown for 3 days under basal (pH 7.4) conditions, lactic acidosis (pH 6.7 + lactate) or in the presence of 10 ng/mL TGFβ1. The basal level of each gene expression was set to 1, and the other results are normalized to this value. 18S ribosomal RNA was used as the reference gene. Bars represent the mean ± SEM of three independent experiments (*n* = 3 replicates each) from the three cell lines. Values of *p* were determined by Tukey’s test.

We next observed that the exposure to lactic acidic conditions in vitro induced the acquisition of a pro-inflammatory and pro-fibrotic phenotype by acADSCs. Indeed, acADSCs cultured in a pH 6.7 medium in the presence of 10 mM lactate showed a 1.5-fold increase in mRNA expression of *IL1B* and *IL6* genes (i.e., genes encoding the pro-inflammatory cytokines interleukin (IL)-1β and IL-6, respectively) compared to those maintained under basal (pH 7.4) conditions (Figure 6A,B). The acADSC treatment with 10 ng/mL TGFβ1 was able to stimulate *IL1B* and *IL6* gene expression at a similar level to lactic acidosis (Figure 6A,B). In line with the above-described acADSC switch toward a myofibroblastic-like phenotype under lactic acidosis, we observed that acADSCs exposed to pH 6.7 in the presence of 10 mM lactate showed a modest but significant increase in the mRNA expression of *TGFB1* (i.e., gene encoding TGFβ1) compared to acADSCs grown under basal conditions (Figure 6C). A similar trend—even though not significant—was observed for the mRNA expression of *TGFB2* (i.e., gene encoding TGFβ2) (Figure 6D).

### 3.3. Pro-Tumoral Activity of Adipocyte-Committed Subcutaneous Adipose-Derived Stem Cells Exposed to the Acidic Microenvironment

**Figure 7:**
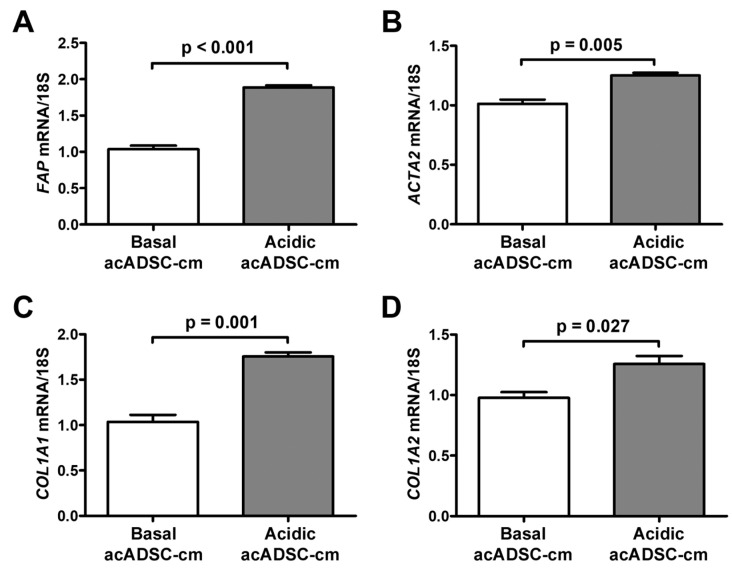
Quantitative real-time PCR analysis for the expression of the fibroblast activation/myofibroblast genes *FAP* (**A**), *ACTA2* (**B**), *COL1A1* (**C**), and *COL1A2* (**D**) in normal human dermal fibroblasts treated for 24 h with conditioned media produced by adipocyte-committed adipose-derived stem cells (acADSCs) grown either under basal (pH 7.4) conditions (basal acADSC-cm) or under lactic acidosis (acidic acADSC-cm). The basal level of each gene expression was set to 1, and the other results are normalized to this value. 18S ribosomal RNA was used as the reference gene. Bars represent the mean ± SEM of three independent experiments (*n* = 3 replicates each) from three cell lines. Values of *p* were determined by the unpaired Student’s *t*-test.

Based on the latter findings, we decided to verify the ability of the acidic acADSCs to promote the reprogramming of naïve stromal cells toward a CAF-like phenotype. At first, we evaluated the effects of the conditioned media collected from acADSCs grown either under basal (pH 7.4) conditions or under lactic acidosis (basal acADSC-cm or acidic acADSC-cm, respectively) on NHDFs. We observed that NHDFs increased the mRNA expression of *FAP* (i.e., gene encoding fibroblast activation protein), *ACTA2*, *COL1A1*, and *COL1A2* genes by approximately 1.5 times when treated with acidic acADSC-cm compared to basal acADSC-cm (Figure 7). These data suggest that acADSCs exposed to a lactic acidic microenvironment, following the AMT process, could be responsible for the activation of quiescent fibroblasts toward a CAF-like phenotype, thereby enabling the hypothesis of their possible involvement in the pro-tumor sustainment.

**Figure 8:**
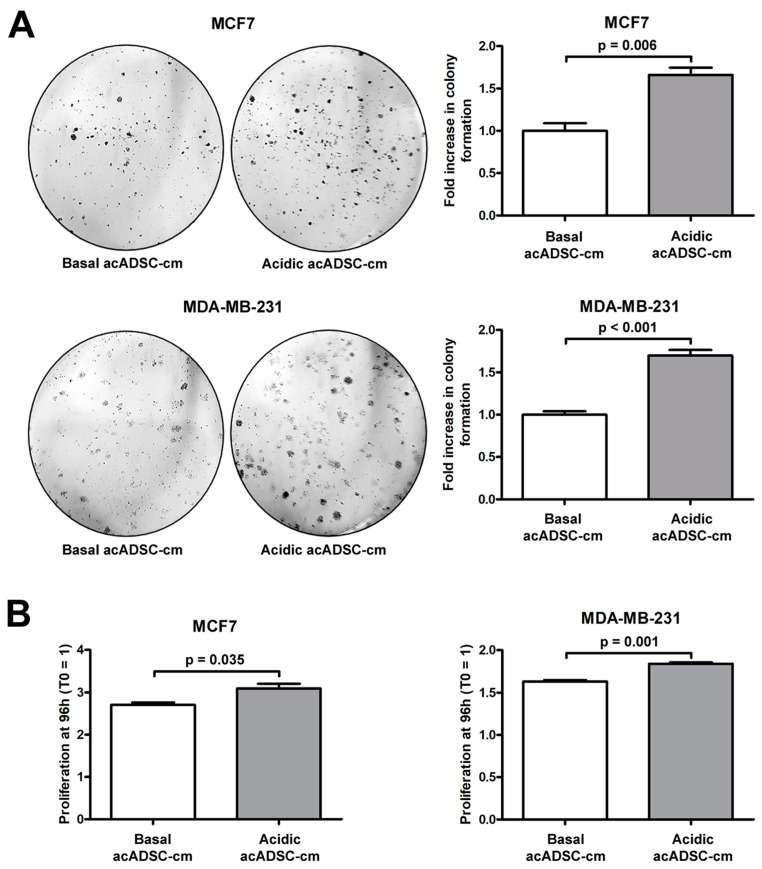
(**A**) Representative images and relative quantification charts of colony formation by MCF7 and MDA-MB-231 cells cultured for 10 days in conditioned media produced by adipocyte-committed adipose-derived stem cells (acADSCs) grown either under basal (pH 7.4) conditions (basal acADSC-cm) or under lactic acidosis (acidic acADSC-cm). (**B**) Proliferation charts of MCF7 and MDA-MB-231 cells cultured for 96 h in basal or acidic acADSC-cm. The cell proliferation rate before the addition of acADSC-cm (T0) was set to 1, and the other results are normalized to this value. Bars represent the mean ± SEM of three independent experiments (*n* = 3 replicates each for colony formation assay, *n* = 10 replicates each for proliferation assay). Values of *p* were determined by unpaired Student’s *t*-test.

Next, we further investigated the pathogenic effects of acidic acADSC-cm, by focusing the attention at this point on breast cancer cells, particularly on the estrogen receptor (ER)-positive MCF7 and the triple-negative MDA-MB-231 cells. Notably, both MCF7 and MDA-MB-231 cells showed a boosted colony formation ability (Figure 8A) and an increased proliferation (Figure 8B) when exposed to acidic acADSC-cm compared to basal acADSC-cm, suggesting the release of pro-tumoral soluble factors by acidic acADSCs in their conditioned media.

**Figure 9:**
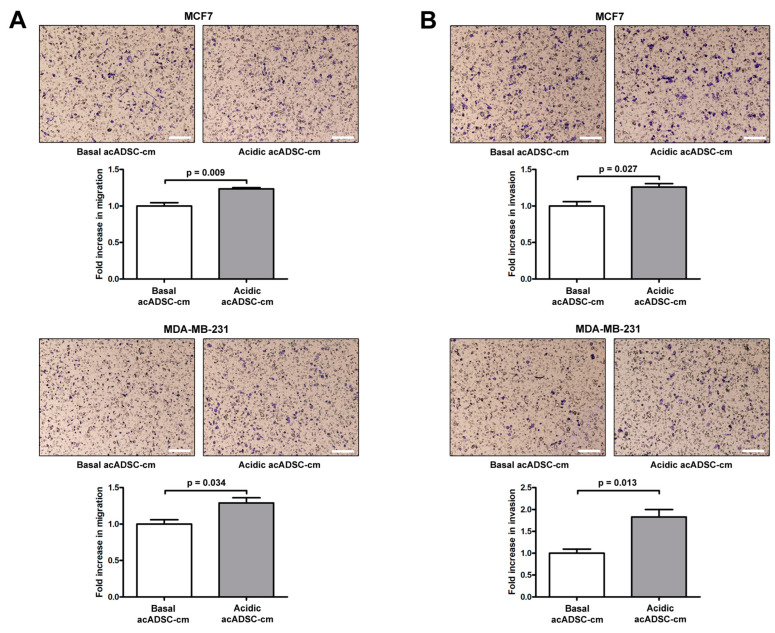
Representative images and relative quantification charts of migration (**A**) and invasion (**B**) assays on MCF7 (top panel) and MDA-MB-231 (bottom panel) cells treated for 24 h with conditioned media produced by adipocyte-committed adipose-derived stem cells (acADSCs) grown either under basal (pH 7.4) conditions (basal acADSC-cm) or under lactic acidosis (acidic acADSC-cm). Images of filters stained with Diff-Quik dye are shown. Scale bar: 250 µm. Migration/invasion of cells treated with basal acADSC-cm was set to 1, and the other results are normalized to this value. Bars represent the mean ± SEM of three independent experiments (*n* = 3 replicates each). Values of *p* were determined by unpaired Student’s *t*-test.

Moreover, both the migratory and invasive abilities of MCF7 and MDA-MB-231 cells were significantly increased following the 24 h treatment with acidic acADSC-cm compared to basal acADSC-cm (Figure 9A,B). More precisely, by treating cancer cells with acidic acADSC-cm, we observed an approximately 1.3-fold increase in the number of MCF7 and MDA-MB-231 cells able to migrate (Figure 9A). In parallel, we detected a 1.3-fold increase in the number of invasive MCF7 cells when treated with the acidic acADSC-cm compared to basal acADSC-cm (Figure 9B). Such an increase was even further evident in the MDA-MB-231 cell line, where the number of invading cells was 1.8 times higher following acidic the acADSC-cm treatment than the basal acADSC-cm (Figure 9B).

**Figure 10:**
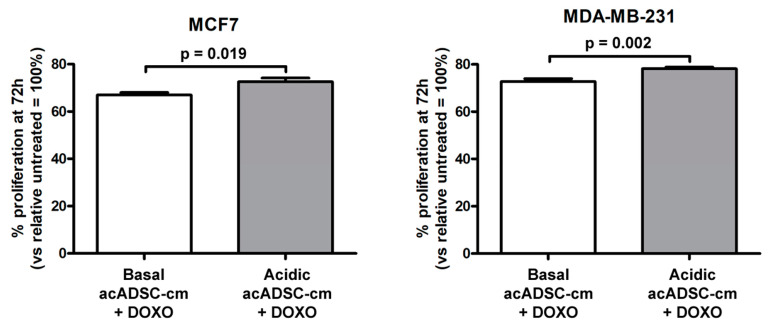
Proliferation charts of MCF7 and MDA-MB-231 cells treated for 72 h, in the presence or absence of doxorubicin (DOXO), with conditioned media from adipocyte-committed adipose-derived stem cells (acADSCs) grown either under basal (pH 7.4) conditions (basal acADSC-cm) or under lactic acidosis (acidic acADSC-cm). Cell proliferation in the absence of DOXO (i.e., untreated) was set to 100%, and the other results are normalized to this value. Bars represent the mean ± SEM of three independent experiments (*n* = 10 replicates each). Values of *p* were determined by unpaired Student’s *t*-test.

Finally, by treating cancer cells with doxorubicin, we observed a slight but significant increase in cell survival, revealed by the higher rate of proliferation of both MCF7 and MDA-MB-231 cells exposed to a conditioned medium collected from the acidosis-exposed acADSCs compared to that of acADSCs grown in a standard medium (Figure 10).

Overall, these findings suggest a potential pro-tumoral effect of acADSCs exposed to an acidic microenvironment to promote the CAF differentiation of standard fibroblasts and support tumor cell proliferation, invasion, and drug resistance.

## 4. Discussion

The TME is a heterogeneous ecosystem composed of multiple cell types including the cells of the immune system, vasculature, and stromal cells, all reprogrammed in favor of tumor sustainment. Extracellular matrix and soluble factors produced by both tumor and host stromal cells are also important elements of the TME, as well as hypoxia and acidosis, which are able to alter and reprogram almost every cell and even non-cellular components of the TME in a pro-tumoral way [32]. Focusing on the biochemical aspects of the TME, to date, much evidence has been provided about the crucial role exerted by hypoxia and acidosis in tumor progression, which made increasingly evident the intense alliance between them and cancer cells, with the common goal of tumor advancement and disease progression [8].

Extracellular acidosis has recently been included in the hallmarks of cancer. Indeed, in the last two decades, the scientific community realized that it represents a peculiar trait of most solid tumors fostering aggressive features of cancer cells and disease progression. This microenvironmental condition arises from the combination of typical features of the tumor tissues, i.e., the boosted cancer glycolytic metabolism with the subsequent release of lactic acid in the extracellular milieu, the impaired drainage by the lymphatic system, and the high interstitial pressure characterizing cancer tissues [2,4]. Extensive literature demonstrates how extracellular lactic acidosis sustains tumor progression by acting either directly on cancer cells, or indirectly, by reprogramming the stromal component within the tumor mass to guarantee cancer cell proliferation and survival even under prohibitive and hostile conditions. Briefly, the acidic TME promotes, on the one hand, the acquisition by cancer cells of high plasticity that renders them extremely adaptable to various scenarios they may encounter [10]; on the other one, extracellular acidosis is able to induce a pro-tumoral reprogramming in the stromal cells, for instance, by altering the capacity of natural and adaptive immune cells to face with the tumor [5,7,17], or by remodeling the vasculature rendering vessels more permeant and subsequently permissive to cancer cell intra/extravasation [33], or further, by inducing the generation of CAFs with all the pro-tumoral activity they are endowed with [12]. What is still not known, is the effect that the acidic TME may exert on the adipocyte compartment.

Here, we demonstrated for the first time that the acidic TME induces the AMT process in adipocytes, fueling the generation of CAF-like cells sustaining the aggressiveness of breast cancer cells. Briefly, we demonstrated that acADSCs grown under lactic acidosis conditions lose the adipocyte differentiation markers FABP4, C/EBPα, adiponectin, and perilipin-1 while acquiring the myofibroblast markers α-SMA, COL1A1, and COL1A2. Such a phenotypic switch driven by lactic acidosis was accompanied by a modest but significant increase in the mRNA expression of the pro-inflammatory cytokines IL-6, IL-1β, and TGFβ1, which in turn could account at least in part for the ability of acid-adapted acADSCs to induce the activation of normal fibroblasts. As recently reviewed [34,35], lactate can serve as a pro- or anti-inflammatory mediator, inducing pleiotropic effects on the inflammatory process. Indeed, lactate could exert differential effects depending not only on the pathological process studied, but also on the cellular metabolic status, and the cell type analyzed. For instance, lactate is pro-inflammatory in endothelial cells and fibroblasts, while it exerts both pro- and anti-inflammatory activity on immune cells depending on the immunophenotypes analyzed. Moreover, the complexity of this scenario increases considering that lactate and H^+^ (i.e., the single components of lactic acidosis) may also exert differential effects in the inflammatory microenvironment, as reviewed by Certo and colleagues [36]. Contextualizing our data in this setting, we did not wonder that lactic acidosis is able to stimulate the production by adipocytes of pro-inflammatory molecules that could account for the fibroblast activation and generation of CAFs with pro-tumoral activity. On the other hand, by reducing the anti-cancer immune response and inducing the pro-tumoral/anti-inflammatory M2 macrophage polarization, lactic acidosis seems to strongly favor cancer progression [36]. It is noteworthy that the conditioned medium of acADSCs exposed to the acidic condition was also able to induce breast cancer cell proliferation, migration, invasion, and drug resistance. Therefore, the identification of the main players of such a pro-tumoral secretome would be of extreme interest in order to develop new anti-cancer strategies.

The peritumoral adipocytes undergo profound reprogramming toward CAAs, especially in those tumors that are closely related to adipose tissue, such as breast, colorectal, and ovarian cancers [37]. Particular attention is dedicated to breast cancer, where the adipose tissue represents the main component. CAAs, in contrast to mature adipocytes, are characterized by a loss of lipid droplets accompanied by a decreased expression of adipocyte differentiation markers [27,38]. Moreover, CAAs may mediate multiple paracrine, juxtacrine, and endocrine signaling, sustaining tumor growth, metastasis, and drug resistance. Then, their pro-tumoral functions may pass through their ability to reprogram cancer metabolism. The disaggregation process of the lipid droplets occurring in adipocyte-to-CAA transformation frees and renders exogenous free fatty acids and high-energy metabolites available to cancer cells, providing them with a precious energy source for their expansion [28,39]. In turn, tumor cells induce lipolysis in adipocytes and promote their transformation toward CAAs [40]. In line with such considerations, our data enable the speculation that the acidic TME may induce the AMT process in which adipocytes are deprived of lipid droplets and, consequently, free fatty acids are freed and made available to either cancer or stromal cells as an important energy source to sustain the overall tumor progression. Interestingly, even pro-tumoral M2 macrophages, induced by a lactic acid-enriched microenvironment, mainly rely on the uptake and subsequent oxidation of free fatty acids freed in the surrounding milieu [41,42]. Evidence already reported the existence of lipid transferring from peritumoral adipocytes to cancer cells, which in turn exploit this high-energetic metabolic source to sustain not only their growth but also to remodel the stromal components to be more permissive to cancer dissemination [43]. The secretome released by CAAs also sustains disease advancement. For instance, it was demonstrated that CCL2/monocyte chemoattractant protein-1, CCL5, IL-1β, IL-6, tumor necrosis factor α, vascular endothelial growth factor, and leptin released by CAAs in the TME promote the proliferation, and dissemination of breast cancer cells [29,44,45]. Furthermore, CAAs induce resistance to chemotherapy, radiation therapy, immunotherapy, and hormone therapy in breast cancer [27], and affect tumor extracellular matrix remodeling and adipose tissue vascularization, leading to the generation of hypoxic and fibrotic conditions that in turn mediate the epithelial-to-mesenchymal transition process in breast cancer cells [46].

Notably, recent evidence suggested that CAAs at the invasive front of the tumor mass undergo the AMT process consisting of the phenotypic switch of adipocytes toward myofibroblast/CAF-like cells endowed with all the pro-tumoral features typical of CAFs [31,47]. Thereby, the AMT, by fueling the CAF compartment, becomes a pro-tumoral process itself.

## 5. Conclusions

Overall, this study highlights that extracellular acidosis may reprogram adipocytes toward pro-tumoral CAF-like cells, which are also able to recruit CAF from quiescent fibroblasts. As a result, the acidic TME could fuel the CAF compartment within the tumor mass, thereby promoting disease advancement. A limiting aspect of this study is that lactic acidosis has only been evaluated in vitro as a unique entity, without determining the single contributions that lactate and acidosis per se could exert in the AMT process. In particular, it would be interesting to further investigate the possible involvement in this phenomenon of lactate transporters (e.g., monocarboxylate transporters MCT1-4 and sodium-dependent transporters SMCT1 and SMCT2) and receptors (e.g., G protein-coupled receptors GPR81 and GPR132) together with the intracellular signaling triggered upon lactate binding and cellular uptake. Another important aspect that needs to be further investigated is whether the lactic acidosis-induced AMT could either be partially or fully reverted when basal pH conditions are restored. These points deserve a dedicated mechanistic study in which the molecular mechanisms leading to AMT need to be identified, to eventually reveal the potential therapeutic targets to counteract tumor progression.

## Figures and Tables

**Table 1:** Predesigned oligonucleotide primer pairs used for SYBR green real-time PCR.

Gene	Assay ID	Catalog No.
*FABP4*	Hs_FABP4_1_SG	QT00055517
*CEBPA*	Hs_CEBPA_1_SG	QT00203357
*PPARG*	Hs_PPARG_1_SG	QT00029841
*ADIPOQ*	Hs_ADIPOQ_1_SG	QT02409862
*PLIN1*	Hs_PLIN1_1_SG	QT00017486
*ACTA2*	Hs_ACTA2_1_SG	QT00088102
*COL1A1*	Hs_COL1A1_1_SG	QT00037793
*COL1A2*	Hs_COL1A2_1_SG	QT00072058
*IL1B*	Hs_IL1B_1_SG	QT00021385
*IL6*	Hs_IL6_1_SG	QT00083720
*TGFB1*	Hs_TGFB1_1_SG	QT00000728
*TGFB2*	Hs_TGFB2_1_SG	QT00025718
*FAP*	Hs_FAP_1_SG	QT00074963

## Data Availability

All relevant data are included within the manuscript and the supplementary materials.

## Supplementary Materials

The following supporting information can be downloaded at: https://www.mdpi.com/article/10.3390/cells12060939/s1, Figure S1: Cell viability quantification of adipose-derived stem cells cultured at different pH ranges; Figure S2: Analysis of cell morphology and expression of adipocytic and myofibroblastic markers in adipocyte-committed adipose-derived stem cells (acADSCs) cultured under basal conditions (pH 7.4) or in the presence of 10 mM lactate (pH 7.4 + lactate); Figure S3: Analysis of the protein expression of the lactate receptor GPR81 in acADSCs cultured under different experimental conditions.

## References

[1] Reshetnyak Y.K., Yao L., Zheng S., Kuznetsov S., Engelman D.M., Andreev O.A. (2011). Measuring Tumor Aggressiveness and Targeting Metastatic Lesions with Fluorescent PHLIP. Mol. Imaging Biol..

[2] Vaupel P., Multhoff G. (2021). Revisiting the Warburg Effect: Historical Dogma *versus* Current Understanding. J. Physiol..

[3] Liu C., Jin Y., Fan Z. (2021). The Mechanism of Warburg Effect-Induced Chemoresistance in Cancer. Front. Oncol..

[4] Vaupel P. (2004). Tumor Microenvironmental Physiology and Its Implications for Radiation Oncology. Semin. Radiat. Oncol..

[5] Corbet C., Feron O. (2017). Tumour Acidosis: From the Passenger to the Driver’s Seat. Nat. Rev. Cancer.

[6] De la Cruz-López K.G., Castro-Muñoz L.J., Reyes-Hernández D.O., García-Carrancá A., Manzo-Merino J. (2019). Lactate in the Regulation of Tumor Microenvironment and Therapeutic Approaches. Front. Oncol..

[7] Fischbeck A.J., Ruehland S., Ettinger A., Paetzold K., Masouris I., Noessner E., Mendler A.N. (2020). Tumor Lactic Acidosis: Protecting Tumor by Inhibiting Cytotoxic Activity Through Motility Arrest and Bioenergetic Silencing. Front. Oncol..

[8] Andreucci E., Peppicelli S., Ruzzolini J., Bianchini F., Calorini L. (2022). Physicochemical Aspects of the Tumour Microenvironment as Drivers of Vasculogenic Mimicry. Cancer Metastasis Rev..

[9] Webb B.A., Chimenti M., Jacobson M.P., Barber D.L. (2011). Dysregulated PH: A Perfect Storm for Cancer Progression. Nat. Rev. Cancer.

[10] Andreucci E., Peppicelli S., Ruzzolini J., Bianchini F., Biagioni A., Papucci L., Magnelli L., Mazzanti B., Stecca B., Calorini L. (2020). The Acidic Tumor Microenvironment Drives a Stem-like Phenotype in Melanoma Cells. J. Mol. Med..

[11] Noren Hooten N., Yáñez-Mó M., DeRita R., Russell A., Quesenberry P., Ramratnam B., Robbins P.D., Di Vizio D., Wen S., Witwer K.W. (2020). Hitting the Bullseye: Are Extracellular Vesicles on Target?. J. Extracell. Vesicles.

[12] Peppicelli S., Bianchini F., Toti A., Laurenzana A., Fibbi G., Calorini L. (2015). Extracellular Acidity Strengthens Mesenchymal Stem Cells to Promote Melanoma Progression. Cell Cycle.

[13] Pavlides S., Whitaker-Menezes D., Castello-Cros R., Flomenberg N., Witkiewicz A.K., Frank P.G., Casimiro M.C., Wang C., Fortina P., Addya S. (2009). The Reverse Warburg Effect: Aerobic Glycolysis in Cancer Associated Fibroblasts and the Tumor Stroma. Cell Cycle.

[14] Bonuccelli G., Avnet S., Grisendi G., Salerno M., Granchi D., Dominici M., Kusuzaki K., Baldini N. (2014). Role of Mesenchymal Stem Cells in Osteosarcoma and Metabolic Reprogramming of Tumor Cells. Oncotarget.

[15] Andreucci E., Margheri F., Peppicelli S., Bianchini F., Ruzzolini J., Laurenzana A., Fibbi G., Bruni C., Bellando-Randone S., Guiducci S. (2021). Glycolysis-Derived Acidic Microenvironment as a Driver of Endothelial Dysfunction in Systemic Sclerosis. Rheumatology.

[16] García-Caballero M., Sokol L., Cuypers A., Carmeliet P. (2022). Metabolic Reprogramming in Tumor Endothelial Cells. Int. J. Mol. Sci..

[17] Erra Díaz F., Dantas E., Geffner J. (2018). Unravelling the Interplay between Extracellular Acidosis and Immune Cells. Mediat. Inflamm..

[18] Wu H., Estrella V., Beatty M., Abrahams D., El-Kenawi A., Russell S., Ibrahim-Hashim A., Longo D.L., Reshetnyak Y.K., Moshnikova A. (2020). T-Cells Produce Acidic Niches in Lymph Nodes to Suppress Their Own Effector Functions. Nat. Commun..

[19] Calcinotto A., Filipazzi P., Grioni M., Iero M., De Milito A., Ricupito A., Cova A., Canese R., Jachetti E., Rossetti M. (2012). Modulation of Microenvironment Acidity Reverses Anergy in Human and Murine Tumor-Infiltrating T Lymphocytes. Cancer Res..

[20] Gottfried E., Kunz-Schughart L.A., Ebner S., Mueller-Klieser W., Hoves S., Andreesen R., Mackensen A., Kreutz M. (2006). Tumor-Derived Lactic Acid Modulates Dendritic Cell Activation and Antigen Expression. Blood.

[21] Onuora S. (2015). Adipocyte–Myofibroblast Transition: Linking Intradermal Fat Loss to Skin Fibrosis in SSc. Nat. Rev. Rheumatol..

[22] Marangoni R.G., Korman B.D., Wei J., Wood T.A., Graham L.V., Whitfield M.L., Scherer P.E., Tourtellotte W.G., Varga J. (2015). Myofibroblasts in Murine Cutaneous Fibrosis Originate From Adiponectin-Positive Intradermal Progenitors: Adipocyte-Myofibroblast Transition in Skin Fibrosis. Arthritis Rheumatol..

[23] Marangoni R.G., Korman B., Varga J. (2020). Adipocytic Progenitor Cells Give Rise to Pathogenic Myofibroblasts: Adipocyte-to-Mesenchymal Transition and Its Emerging Role in Fibrosis in Multiple Organs. Curr. Rheumatol. Rep..

[24] Rosa I., Romano E., Fioretto B.S., Matucci-Cerinic M., Manetti M. (2021). Adipose-Derived Stem Cells: Pathophysiologic Implications *vs* Therapeutic Potential in Systemic Sclerosis. World J. Stem Cells.

[25] Mukherjee A., Bilecz A.J., Lengyel E. (2022). The Adipocyte Microenvironment and Cancer. Cancer Metastasis Rev..

[26] Rybinska I., Mangano N., Tagliabue E., Triulzi T. (2021). Cancer-Associated Adipocytes in Breast Cancer: Causes and Consequences. Int. J. Mol. Sci..

[27] Zhao C., Wu M., Zeng N., Xiong M., Hu W., Lv W., Yi Y., Zhang Q., Wu Y. (2020). Cancer-Associated Adipocytes: Emerging Supporters in Breast Cancer. J. Exp. Clin. Cancer Res..

[28] Wu Q., Li B., Li Z., Li J., Sun S., Sun S. (2019). Cancer-Associated Adipocytes: Key Players in Breast Cancer Progression. J. Hematol. Oncol..

[29] Lapeire L., Hendrix A., Lambein K., Van Bockstal M., Braems G., Van Den Broecke R., Limame R., Mestdagh P., Vandesompele J., Vanhove C. (2014). Cancer-Associated Adipose Tissue Promotes Breast Cancer Progression by Paracrine Oncostatin M and Jak/STAT3 Signaling. Cancer Res..

[30] D’Esposito V., Ambrosio M.R., Giuliano M., Cabaro S., Miele C., Beguinot F., Formisano P. (2020). Mammary Adipose Tissue Control of Breast Cancer Progression: Impact of Obesity and Diabetes. Front. Oncol..

[31] Zhu Q., Zhu Y., Hepler C., Zhang Q., Park J., Gliniak C., Henry G.H., Crewe C., Bu D., Zhang Z. (2022). Adipocyte Mesenchymal Transition Contributes to Mammary Tumor Progression. Cell Rep..

[32] Neophytou C.M., Panagi M., Stylianopoulos T., Papageorgis P. (2021). The Role of Tumor Microenvironment in Cancer Metastasis: Molecular Mechanisms and Therapeutic Opportunities. Cancers.

[33] Andreucci E., Ruzzolini J., Bianchini F., Versienti G., Biagioni A., Lulli M., Guasti D., Nardini P., Serratì S., Margheri F. (2022). MiR-214-Enriched Extracellular Vesicles Released by Acid-Adapted Melanoma Cells Promote Inflammatory Macrophage-Dependent Tumor Trans-Endothelial Migration. Cancers.

[34] Baltazar F., Afonso J., Costa M., Granja S. (2020). Lactate Beyond a Waste Metabolite: Metabolic Affairs and Signaling in Malignancy. Front. Oncol..

[35] Manosalva C., Quiroga J., Hidalgo A.I., Alarcón P., Anseoleaga N., Hidalgo M.A., Burgos R.A. (2022). Role of Lactate in Inflammatory Processes: Friend or Foe. Front. Immunol..

[36] Certo M., Llibre A., Lee W., Mauro C. (2022). Understanding Lactate Sensing and Signalling. Trends Endocrinol. Metab..

[37] Yao H., He S. (2021). Multi-faceted Role of Cancer-associated Adipocytes in the Tumor Microenvironment (Review). Mol. Med. Rep..

[38] Choi J., Cha Y.J., Koo J.S. (2018). Adipocyte Biology in Breast Cancer: From Silent Bystander to Active Facilitator. Prog. Lipid Res..

[39] Pérez-Escuredo J., Van Hée V.F., Sboarina M., Falces J., Payen V.L., Pellerin L., Sonveaux P. (2016). Monocarboxylate Transporters in the Brain and in Cancer. Biochim. Biophys. Acta.

[40] Yang E., Wang X., Gong Z., Yu M., Wu H., Zhang D. (2020). Exosome-Mediated Metabolic Reprogramming: The Emerging Role in Tumor Microenvironment Remodeling and Its Influence on Cancer Progression. Sig. Transduct. Target. Ther..

[41] Kim J. (2018). Regulation of Immune Cell Functions by Metabolic Reprogramming. J. Immunol. Res..

[42] Zhou H., Yan X., Yu W., Liang X., Du X., Liu Z., Long J., Zhao G., Liu H. (2022). Lactic Acid in Macrophage Polarization: The Significant Role in Inflammation and Cancer. Int. Rev. Immunol..

[43] Wagner M., Bjerkvig R., Wiig H., Melero-Martin J.M., Lin R.-Z., Klagsbrun M., Dudley A.C. (2012). Inflamed Tumor-Associated Adipose Tissue Is a Depot for Macrophages That Stimulate Tumor Growth and Angiogenesis. Angiogenesis.

[44] D’Esposito V., Liguoro D., Ambrosio M.R., Collina F., Cantile M., Spinelli R., Raciti G.A., Miele C., Valentino R., Campiglia P. (2016). Adipose Microenvironment Promotes Triple Negative Breast Cancer Cell Invasiveness and Dissemination by Producing CCL5. Oncotarget.

[45] De Palma M., Biziato D., Petrova T.V. (2017). Microenvironmental Regulation of Tumour Angiogenesis. Nat. Rev. Cancer.

[46] Ribeiro R.J.T., Monteiro C.P.D., Cunha V.F.P.M., Azevedo A.S.M., Oliveira M.J., Monteiro R., Fraga A.M., Príncipe P., Lobato C., Lobo F. (2012). Tumor Cell-Educated Periprostatic Adipose Tissue Acquires an Aggressive Cancer-Promoting Secretory Profile. Cell. Physiol. Biochem..

[47] Rybinska I., Agresti R., Trapani A., Tagliabue E., Triulzi T. (2020). Adipocytes in Breast Cancer, the Thick and the Thin. Cells.

