# Supplementary material for: Extracellular Lactic Acidosis of the Tumor Microenvironment Drives Adipocyte-to-Myofibroblast Transition Fueling the Generation of Cancer-Associated Fibroblasts
Source: Cells. 2023 Mar 20;12(6):939. doi: 10.3390/cells12060939 (PMC10046917; doi:10.3390/cells12060939)
Supplement: Supplementary file 1 [file cells-12-00939-s001.zip › Supplementary Figure S1.pdf]

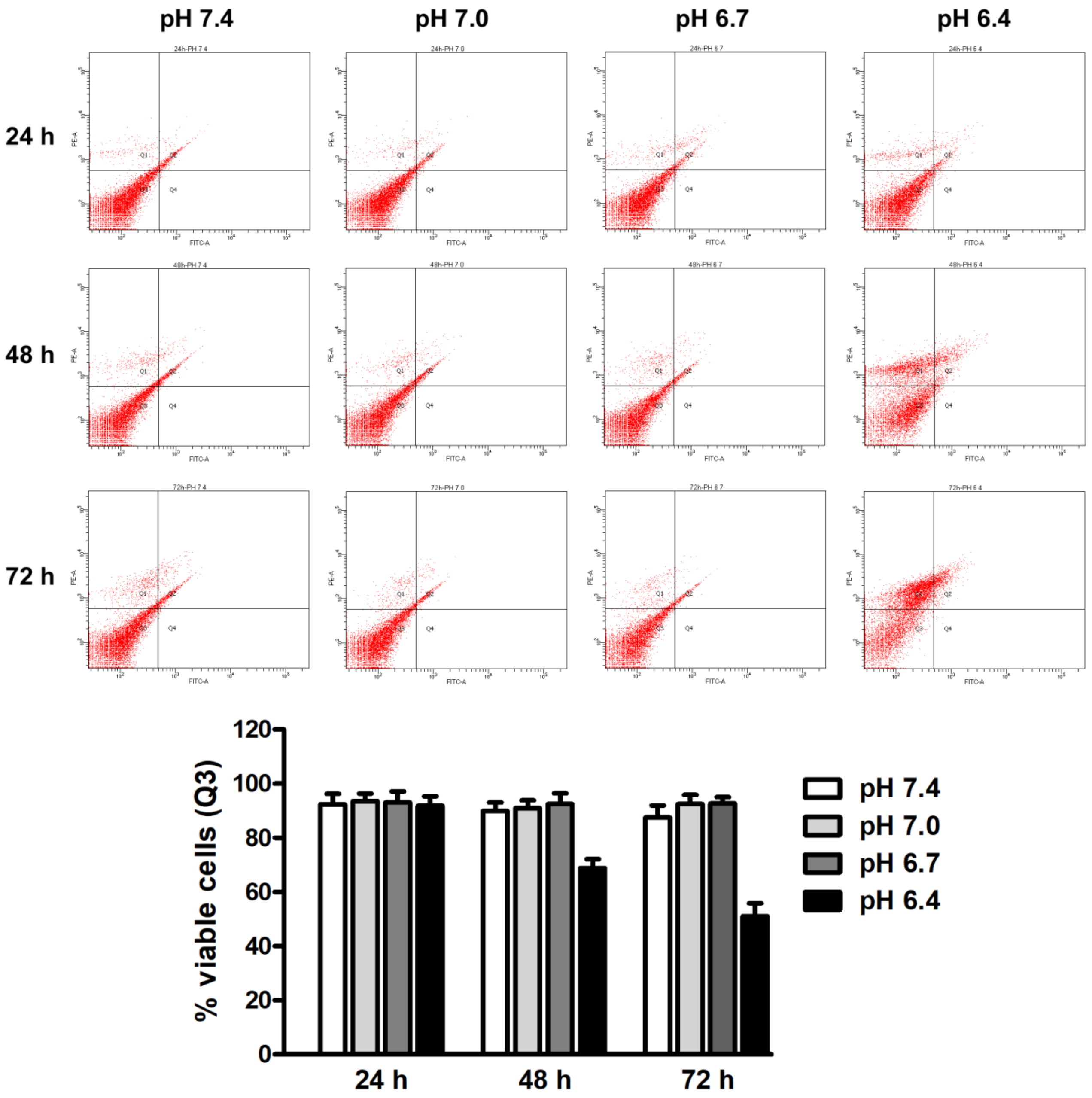

Supplementary Figure S1. Representative flow cytometer plots of annexin V/propidium iodide staining with cell viability quantification of adipose-derived stem cells (ADSCs) cultured for 24, 48, and 72 h at different pH ranges (i.e., pH 7.4, pH 7.0, pH 6.7, and pH 6.4). Bars represent the mean ± SEM of triplicate determinations from three cell lines. A substantial reduction in the percentage of viable cells (Q3) was observed in ADSCs cultured at pH 6.4 for 48 and 72 h.
