# Supplementary material for: Extracellular Lactic Acidosis of the Tumor Microenvironment Drives Adipocyte-to-Myofibroblast Transition Fueling the Generation of Cancer-Associated Fibroblasts
Source: Cells. 2023 Mar 20;12(6):939. doi: 10.3390/cells12060939 (PMC10046917; doi:10.3390/cells12060939)
Supplement: Supplementary file 1 [file cells-12-00939-s001.zip › Supplementary Figure S2.pdf]

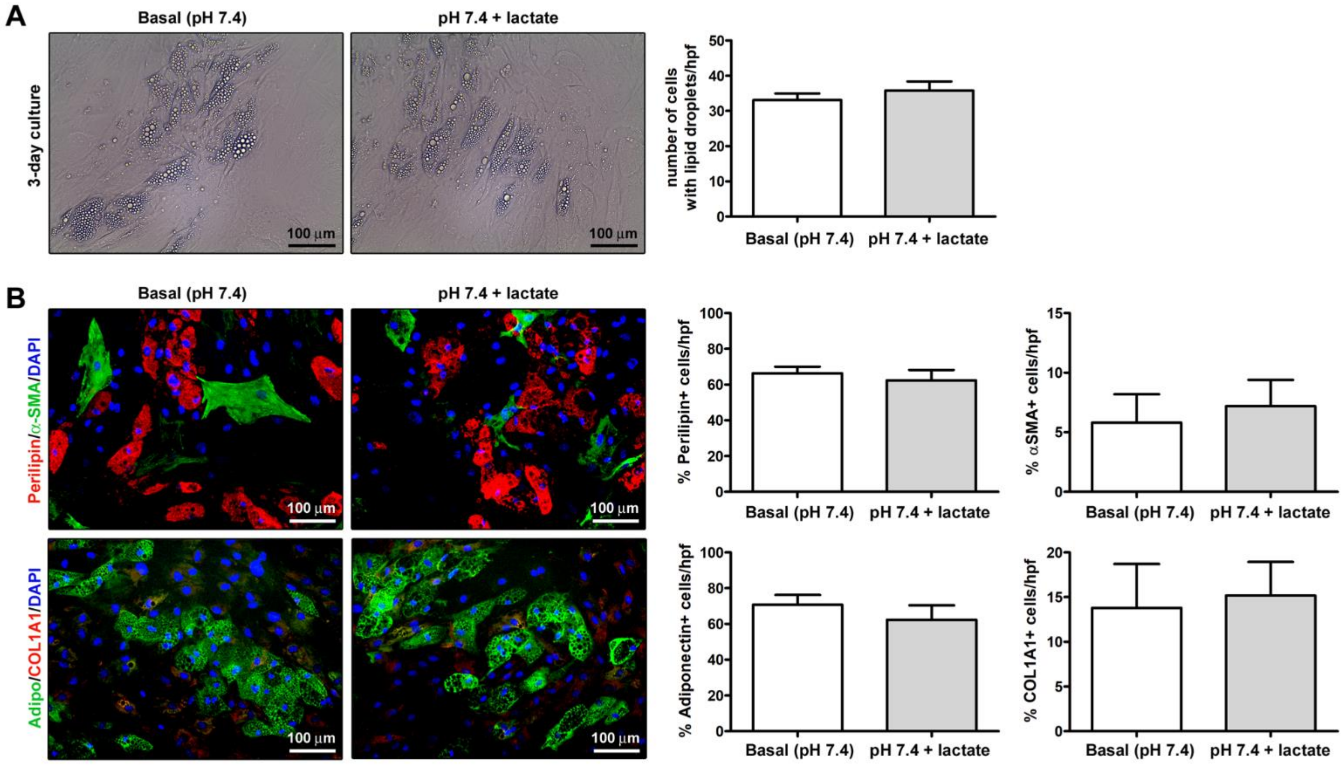

Supplementary Figure S2. (**A**) Representative phase-contrast photomicrographs of adipocyte-committed adipose-derived stem cells (acADSCs) cultured under basal conditions (pH 7.4) or in the presence of 10 mM lactate (pH 7.4 + lactate) for 3 days. Scale bar: 100 μm. Bars represent the mean ± SEM of the number of cells with intracytoplasmic lipid droplets/high-power field (hpf). (**B**) Immunofluorescence analysis for the expression of the adipocytic markers perilipin-1 (red, upper panels) and adiponectin (green, lower panels), and the myofibroblastic markers α-SMA (green, upper panels) and COL1A1 (red, lower panels) in acADSCs grown for 3 days under basal (pH 7.4) conditions or in the presence of 10 mM lactate (pH 7.4 + lactate). Nuclei are stained blue with DAPI. Scale bar: 100 μm. Bars represent the mean ± SEM of the percentage of immunopositive cells/hpf. No statistically significant difference was detected for the different parameters by unpaired Student's t-test.
