# Supplementary material for: Extracellular Lactic Acidosis of the Tumor Microenvironment Drives Adipocyte-to-Myofibroblast Transition Fueling the Generation of Cancer-Associated Fibroblasts
Source: Cells. 2023 Mar 20;12(6):939. doi: 10.3390/cells12060939 (PMC10046917; doi:10.3390/cells12060939)
Supplement: Supplementary file 1 [file cells-12-00939-s001.zip › Supplementary Figure S3.pdf]

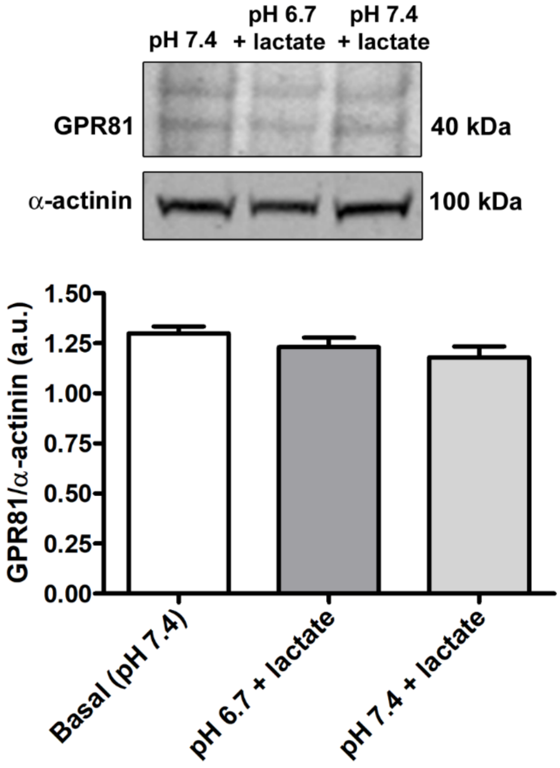

Supplementary Figure S3. Representative bands of western blot analysis and relative quantification chart of the expression of the lactate receptor GPR81 in adipocyte-committed adipose-derived stem cells grown for 3 days under basal conditions (pH 7.4), lactic acidosis (pH 6.7 + 10 mM lactate), or in the presence of 10 mM lactate (pH 7.4 + lactate). α-actinin was measured as a loading control for normalization. Molecular weight values (kDa) are indicated. Bars represent the mean ± SEM of optical density in arbitrary units (a.u.). No statistically significant difference in protein levels of GPR81 was detected by Tukey’s test.
